# Effectiveness of a clinical decision support system for hypertension management in primary care: study protocol for a pragmatic cluster-randomized controlled trial

**DOI:** 10.1186/s13063-022-06374-x

**Published:** 2022-05-16

**Authors:** Jiali Song, Xiuling Wang, Bin Wang, Yan Gao, Jiamin Liu, Haibo Zhang, Xi Li, Jing Li, Ji-Guang Wang, Jun Cai, Jeph Herrin, Jane Armitage, Harlan M. Krumholz, Xin Zheng, on behalf of the LIGHT Collaborative Group

**Affiliations:** 1 National Clinical Research Center for Cardiovascular Diseases, State Key Laboratory of Cardiovascular Disease, Chinese Academy of Medical Sciences and Peking Union Medical College, Fuwai Hospital, National Center for Cardiovascular Diseases, 167 Beilishi Road, Beijing, 100037 China; 2 The Shanghai Institute of Hypertension, Ruijin Hospital, Shanghai Jiaotong University School of Medicine, Shanghai, China; 3 Hypertension Center, State Key Laboratory of Cardiovascular Disease, Chinese Academy of Medical Sciences and Peking Union Medical College, Fuwai Hospital, Beijing, China; 4 Section of Cardiovascular Medicine, Department of Internal Medicine, Yale University School of Medicine, New Haven, Connecticut USA; 5 Clinical Trial Service Unit and Epidemiological Studies Unit, Nuffield Department of Population Health, University of Oxford, Richard Doll Building, Old Road Campus, Roosevelt Drive, Oxford, OX3 7LF UK; 6 MRC Population Health Research Unit, Nuffield Department of Population Health, University of Oxford, Oxford, UK; 7 Center for Outcomes Research and Evaluation, Yale-New Haven Hospital, New Haven, Connecticut USA; 8 Department of Health Policy and Management, Yale School of Public Health, New Haven, Connecticut USA; 9 National Clinical Research Center for Cardiovascular Diseases, Shenzhen, Coronary Artery Disease Center, Fuwai Hospital Chinese Academy of Medical Sciences, Shenzhen, China

**Keywords:** Hypertension, Clinical decision support system, Pragmatic trial

## Abstract

**Background:**

Clinical decision support systems (CDSS) are low-cost, scalable tools with the potential to improve guideline-based antihypertensive treatment in primary care, but their effectiveness needs to be tested, especially in low- and middle-income countries such as China.

**Methods:**

The Learning Implementation of Guideline-based decision support system for Hypertension Treatment (LIGHT) trial is a pragmatic, four-stage, cluster-randomized trial conducted in 94 primary care sites in China. For each city-based stage, sites are randomly assigned to either implementation of the CDSS for hypertension management (which guides doctors’ treatment recommendations based on measured blood pressure and patient characteristics), or usual care. Patients are enrolled during the first 3 months after site randomization and followed for 9 months. The primary outcome is the proportion of hypertension management visits at which guideline-based treatment is provided.

In a nested trial conducted within the CDSS, with the patient as the unit of randomization, the LIGHT-ACD trial, patients are randomized to receive different initial mono- or dual-antihypertensive therapy. The primary outcome of the LIGHT-ACD trial is the changes in blood pressure.

**Discussion:**

The LIGHT trial will provide evidence on the effectiveness of a CDSS for improving guideline adherence for hypertension management in primary care in China. The nested trial, the LIGHT-ACD trial, will provide data on the effect of different initial antihypertensive regimens for blood pressure management in this setting.

**Trial registration:**

ClinicalTrials.gov, identifier: LIGHT (NCT03636334) and LIGHT-ACD (NCT03587103). Registered on 3 July 2018.

**Supplementary Information:**

The online version contains supplementary material available at 10.1186/s13063-022-06374-x.

## Introduction

Hypertension is the leading modifiable risk factor for death globally [1]. Over the past decades, the number of individuals with hypertension is estimated to have increased by 90%, with the majority of the increase occurring in low- and middle-income countries (LMICs) [2]. In China, an estimated 244.5 million adults have hypertension, and only about 15% of these individuals have adequate blood pressure control, resulting in major health and economic burdens [3].

Improving the performance of primary care doctors, who play a key role in managing hypertension, and ensuring their adherence to guideline-recommended antihypertensive treatments are public health priorities in China [4]. Despite the decade-long efforts to strengthen the primary care system [5], the underuse of guideline-based antihypertensive treatments persists [6–8]. Moreover, given the substantial gaps in the licensure and education of primary care doctors [9], traditional strategies to empower their practices including educational training are resource-intensive to implement and may only yield modest effects [10]. A clinical decision support system (CDSS), characterized by generating recommendations through computerized algorithms, may circumvent these barriers to adequate management of hypertension [4, 11]. However, evidence of CDSS enhancing guideline adherent prescribing in hypertension is limited, especially in LMICs [12, 13]. Most studies have focused on assessing its effect on blood pressure reduction and have shown mixed results [14–16]. Understanding the effectiveness of CDSS for hypertension management in primary care could provide important information to develop effective strategies for mitigating the burden of hypertension in China and other LMICs.

In addition, in the course of testing the CDSS, there is an opportunity to test different antihypertensive regimens. Blood pressure guidelines endorse these antihypertensive therapies as equivalent even though there is a paucity of head-to-head comparisons [17, 18]. Using the CDSS to promote guideline adherence may facilitate such comparisons since randomization of treatment can be performed within the CDSS each time a decision support recommendation is generated for the patient. Nesting a randomized trial comparing antihypertensive regimens within CDSS will optimize the conduct and efficiency of the nested trial by streamlining the enrolment and follow-up.

Accordingly, our main aim is to conduct a cluster-randomized controlled trial, the Learning Implementation of Guideline-based decision support system for Hypertension Treatment (LIGHT) trial, to assess the effectiveness of a CDSS on improving guideline-concordant antihypertensive treatment and blood pressure control compared to usual care. Within the LIGHT trial, we also conduct a nested trial, the LIGHT-ACD trial, to compare blood pressure changes between different initial guideline-recommended drug regimens.

## Methods

### The LIGHT trial

#### Objectives

The primary objective of the LIGHT trial is to determine whether CDSS is effective in improving the use of guideline-based antihypertensive treatment compared to usual care, and the secondary objectives are to determine its effect on blood pressure changes and blood pressure control.

#### Trial design and setting

**Fig. 1:**
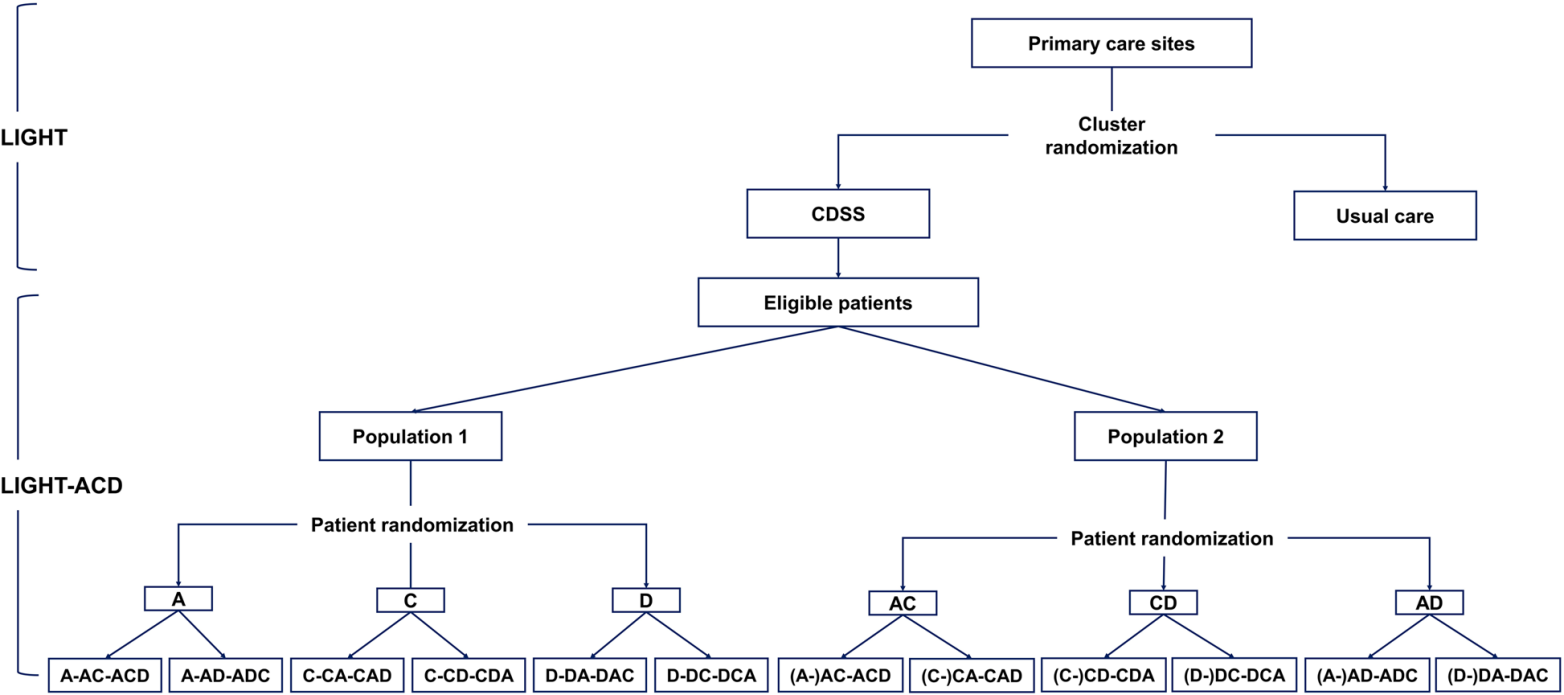
Infrastructure of the LIGHT and LIGHT-ACD trials

**Fig. 2:**
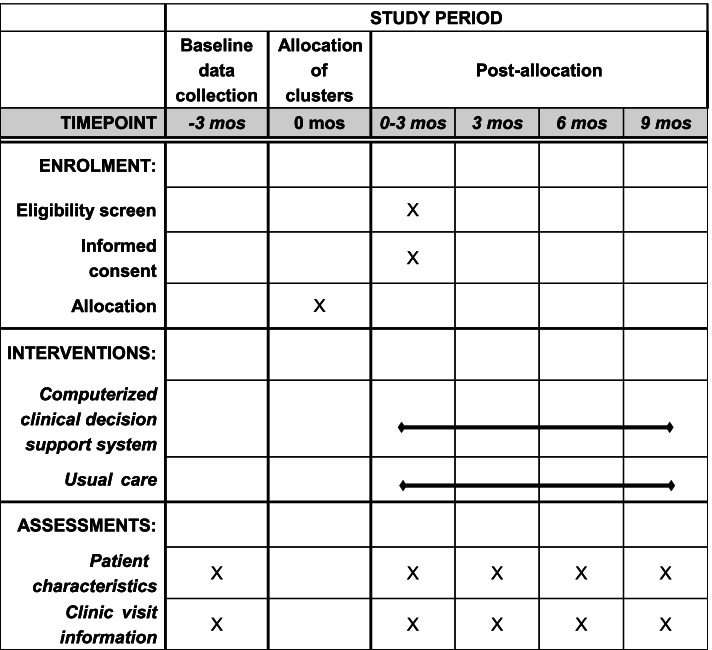
Schedule of enrollment, intervention allocation and assessment of the LIGHT trial using Standard Protocol Items: Recommendations for Interventional Trials (SPIRIT)

The LIGHT trial is a pragmatic, parallel-group, four-stage, cluster-randomized controlled trial assessing the effectiveness of CDSS in primary care in China, with primary care sites (including community health centers, community health stations, and township health centers) as the unit of randomization. In each stage, urban primary care sites are randomized to either implementation of the CDSS for hypertension management or usual care (Fig. 1). During the first 3 months after cluster randomization, patients who attend the clinic for hypertension management are screened for eligibility and if eligible asked to attend the clinic at least every 3 months with data collected at each visit (Fig. 2).

#### Eligibility criteria

In each city-based stage, local coordination centers of the trial invite all primary care sites in their network to participate in the trial. Primary care sites with hypertension clinics are eligible to be included if (1) at least one agent from each of the four classes of antihypertensive medication is available for the prescription [angiotensin-converting enzyme inhibitors (ACEI)/angiotensin receptor blockers (ARB), beta-blockers, calcium channel blockers (CCB), diuretics]; (2) an electronic health record (EHR) is being used for hypertension management providing a structured template for collection of patient data; and (3) at least 100 patients with hypertension are routinely being seen. Cluster drop-out is not expected but might occur if sites voluntarily withdraw during the trial.

Residents aged ≥18 years registered for hypertension treatment at the primary care sites are eligible for the LIGHT trial if they are taking 0–2 classes of antihypertensive medication. Major exclusion criteria include (1) history of coronary heart disease, heart failure, or chronic kidney disease; or (2) intolerance to 2 or more classes of antihypertensive medications; (3) systolic blood pressure (SBP) ≥180 mmHg or diastolic blood pressure (DBP) ≥110 mmHg at the screening visit (see detailed inclusion and exclusion criteria in Table 1). The screening of patients can be automatically performed by the customized EHR. As the informed consent for implementing the CDSS is waived in this trial, doctors can check the eligibility of all patients attending their clinics for hypertension management.

**Table 1:** Inclusion and exclusion criteria of participants for LIGHT and LIGHT-ACD trials

Criteria	LIGHT	LIGHT-ACD
**Inclusion**	Residents aged ≥18 years registered for hypertension treatment at primary care sites	Participants from intervention sites of LIGHT study with systolic blood pressure ≥140 mmHg at the screening visit
	Taking 0–2 classes of antihypertensive medications	Not taking antihypertensive medication or taking only one which was not beta-blocker
**Exclusion**	History of coronary heart disease^a^, heart failure, and chronic kidney disease	Known/diagnosed diabetes mellitus
	Intolerance to 2 or more classes of antihypertensive medications	Intolerance to 1 or more classes of antihypertensive medications
	Secondary hypertension (physician-diagnosed)	Home blood pressure (if available) below 135/85 mmHg
	Systolic blood pressure ≥180 mmHg or diastolic blood pressure ≥110 mmHg at the screening visit	
	Serious medical conditions (e.g., malignant cancer and hepatic dysfunction)	
	Currently in an acute episode of disease	
	Currently pregnant or breastfeeding, or planning a pregnant or breastfeeding during the study	
	Cognitive or communication disorders	

^a^ Including angina, myocardial infarction, coronary artery bypass grafting, percutaneous coronary intervention, >50% stenosis of coronary artery, or positive stress test

#### Allocation

Primary care sites selected are randomized to intervention or control arm (1:1 ratio) using computer-generated random numbers stratified by baseline appropriate antihypertensive treatment rate and site characteristics (see detailed stratifying factors for each stage in Supplement 1). Site randomization is performed centrally, and participating sites will be confirmed by local coordinating centers before randomization. The statistician assigns sites to the randomization strata and uses a computer program to randomly allocate half the sites in each strata to the intervention group; the remaining sites are allocated to the control group. Group allocation is unknown to any other team member before a formal announcement to the investigators and sites. Given the nature of the intervention, doctors and participants cannot be blinded. Data analysts will follow a predefined analysis plan to avoid bias.

#### Intervention

The intervention consists of an EHR-based CDSS providing antihypertensive recommendations generated from information recorded at the visit. The recommendations are based on the Chinese primary care hypertension guidelines [19] but are generally consistent with international guidance [20, 21] and include treatment advice to add one or more of the 4 classes of medication at half or full dose (see an example of CDSS recommendation in Supplement 3). During prescribing, the dosages and frequencies of drugs can be filled in automatically according to the specific agents entered by doctors. To ensure that the intervention is being reliably delivered to doctors, clicking the icon for obtaining CDSS recommendations is designed to be mandatory before prescribing in the EHR. If the doctor decides not to follow the recommendation, a pop-up message stating “your prescription did not match with CDSS recommendation, please re-evaluate the classes/doses/frequencies of drugs.” and prescriptions can be modified accordingly. If the doctor decides not to follow the CDSS recommendation, relevant reasons (e.g., doctor preference or patient refusal) are recorded. In addition, if prescriptions involve contraindicated drugs, under-dosage, or over-dosage, a message warning is also triggered.

The CDSS algorithm was developed by clinicians in the research team based on guidelines for hypertension management [19–21]. After the algorithm was finalized, our information technology (IT) team translated it into computational logic. The logic of the CDSS was tested using simulated patient data to trigger each possibility. The CDSS was further validated by comparing the treatment recommendations provided by CDSS with that by clinicians for specific test cases. The IT team was notified when any discrepancies were found so that the programming errors could be identified. This process was repeated until no errors were observed in all test cases. The CDSS was piloted in two excluded sites. During the pilot test, there were a total of 78 hypertension visits. The mean SBP and DBP were 131.5 mmHg and 76.6 mmHg, respectively. Doctors followed the CDSS recommendations in 52.6% of hypertension visits. The most common reason for the non-compliance was patient refused to follow even though such recommendations had been provided by doctors in the shared decision process (83.8%).

After randomization, doctors in the intervention sites receive face-to-face or online training on the use and interpretation of the CDSS output. In addition, the evidence base of the output is briefly introduced. After the training, the IT team releases a new version of EHR with CDSS available for the intervention sites, doctors are instructed to use the CDSS directly in all hypertension visits thereafter.

#### Control

In control sites, the EHR is used to record the same information, but no antihypertensive recommendation is made. Prescribing decisions are made by doctors based on their knowledge and usual practice.

#### Outcomes

The primary outcome for the LIGHT trial is the proportion of hypertension visits (see Supplement 2 for specification) during which appropriate antihypertensive treatment is prescribed. Appropriate antihypertensive treatment is defined as a prescription compliant with the pre-specified guideline-based recommendations (see Supplement 4 for specifications). The secondary outcomes include the average change in systolic blood pressure, the proportion of those whose blood pressure is controlled at 9 months, and the proportion of hypertension visits with *acceptable* antihypertensive treatment, which is defined as either appropriate antihypertensive treatment (as above) or those treatments with acceptable reasons for failing to titrate antihypertensive treatment. An exploratory outcome is the number of patients experiencing a vascular event defined as a composite of cardiac death, non-fatal stroke, and non-fatal myocardial infarction (Table 2).

**Table 2:** Outcomes of LIGHT and LIGHT-ACD trials

Outcomes	LIGHT	LIGHT-ACD
**Primary**	Appropriate (guideline-based) antihypertensive treatment rate	Absolute change in blood pressure at 9 months of different regimens of initial therapy
**Secondary**	Absolute change of systolic blood pressure at 9 months	Proportion of individuals with blood pressure controlled (SBP <140 mmHg and DBP <90 mmHg) at 9 months
	Blood pressure control rate (defined as SBP <140 mmHg and DBP <90 mmHg) at 9 months	Proportion of individuals with SBP <160 mmHg and DBP <100 mmHg at 9 months
	Acceptable (appropriate/non-appropriate but with proper reasons) antihypertensive treatment rate	Proportion of individuals who received monotherapy^a^, dual therapy, triple therapy, and referral at 9 months
		Proportion of individuals with antihypertensive drug side-effects
		Proportion of individuals transferred to usual care for any reason
**Exploratory**	A composite of cardiac death, non-fatal stroke, and non-fatal myocardial infarction	Absolute change in blood pressure at 9 months of different protocols^a^

Primary and secondary outcomes of LIGHT-ACD were assessed among initiating therapies; exploratory outcomes of LIGHT-ACD were assessed among protocols

*SBP* systolic blood pressure, *DBP* diastolic blood pressure

^a^ Only assessed in Population 1, who are not currently taking any antihypertensive medication with systolic blood pressure 140–159 mmHg, and initiated with monotherapy

#### Data collection and management

To improve the workflow, patient data are collected via the customized EHR, which only collects information necessary for hypertension pharmacological management (Table 3). The EHR performs built-in data checks to verify that the data are complete and meet predefined data ranges and formats. Blood pressure is measured with a validated automated sphygmomanometer (Omron HBP-1300) [22], after at least a 5-min rest in the sitting position. Two blood pressure readings are taken 1–2 min apart, and the average value is recorded. Trained primary care doctors (with the assistance of other trained providers at some sites) are responsible for participant enrolment and follow-up. After enrolment, eligible participants are asked to attend the clinic at least every 3 months for follow-up. As we include participants registered for hypertension treatment at the primary care site in this trial, a substantial proportion of them are expected to be retained. To maximize the retention of participants, they will also be contacted and invited to attend the clinics for collecting data during follow-up.

**Table 3:** Data elements collected in baseline, recruitment, and follow-up

	Baseline	Recruitment	Follow-up
Socio-demographics (age, gender, education, and insurance)	√	√	√
Physical measurements (blood pressure, heart rate, waist, height, and weight)	√	√	√
Self-reported home monitoring blood pressure		√	√
Cardiovascular risk factors	√	√	√
Comorbidities	√	√	√
Hospitalizations		√	√
Adverse events		√	√
Current medications	√	√	√
Medication adherence^a^	√	√	√
Side-effects related to antihypertensive medication		√	√
CDSS recommendations^b^		√	√
Prescriptions	√	√	√
Reasons for not following CDSS^b^		√	√

^**a**^ Adherence to each antihypertensive medication is collected as three classes: regularly, intermittently, and rarely

^b^ Only in the intervention sites of the LIGHT trial

All data are securely transmitted to the central server through automatic electronic transfer and securely stored in an encrypted and password-protected database. Data confidentiality policies on data collection, storage, and analysis have been strictly imposed to ensure the confidentiality of personal information.

#### Data monitoring and quality control

The Trial Steering Committee periodically reviews trial progress and provides advice to the investigators on all aspects of the trial. No interim analyses of intervention effectiveness are planned. Due to the low-risk nature of the trial, a Data Monitoring Committee is not necessary. Similarly, there are no anticipated adverse events and harms from the trial intervention. Any unanticipated problems and adverse events will be reported to the research team and appropriate regulatory bodies.

We randomly select blood pressure values recorded in EHR daily (at least one value per site) and check against the recordings in the automated sphygmomanometer. We also compare the prescription recorded in the EHR with paper/electronic prescription sheets to verify their consistency biweekly. A bespoke website was developed for the research team to monitor the progress and quality of the trial in real time. Through this website, we can review the comparison results of blood pressure values in EHR and sphygmomanometer to monitor the accuracy of documentation in all sites, and any data with invalid values can be searched and traceable corrected. CDSS recommendations can be also viewed via the website to ensure that the CDSS is working as designed. In addition, on-site audits are regularly conducted by the research team to verify that data are generated, documented, and reported in compliance with the protocol.

#### Sample size

As this is a staged pragmatic trial, we have the capacity to add sites as the trial progresses. Initial statistical power was based on the number of sites potentially eligible in stage 1 at the time the power analysis was being conducted; thus, we assumed that at least 10 primary care sites would be randomized to the intervention arm and 10 to the control arm, the baseline appropriate treatment rate would be 55% with maximum type I error of α = 0.05, a moderate intra-site correlation of 0.05, a within-patient correlation of 0.1, and statistical power of 90%, and we needed 3 hypertension visits per patient for 50 patients at each site to detect an 18% absolute difference in the appropriate treatment rate between the two arms. Subsequent enrolment increased the number of participating sites to 94, randomized in 4 stages; under the same assumptions as above, we anticipate that the final sample will provide 90% power to detect a true difference of 4% in the appropriate treatment rate. This difference is close to the average effect of CDSS in improving the process of care in prior studies [23, 24].

#### Statistical analysis

The analyses and reporting of the results will follow the Consolidated Standards of Reporting Trials guidelines for cluster-randomized controlled trials [25]. All the analyses will be performed on an intention-to-treat basis. Multiple imputations by chained equations will be used to account for missing values.

With all comparative outcomes, we will use mixed-effects generalized linear regression models, using logistic or linear response for dichotomous or continuous outcomes, respectively, and including a random effect for the study site. Implementation stages will be treated as strata, with adjustment for calendar time to account for secular trends. The consistency of treatment effects on the primary outcome will be explored in predefined subgroups, including age, gender, education, implementation stage, tertiles of baseline cluster-level appropriate treatment rate, blood pressure level, and use of antihypertensive medications. All statistical tests will be performed using 2-sided tests at the 0.05 level of significance, but the number of tests and *p*-value will be taken into account in the interpretation of the results.

### The LIGHT-ACD trial

#### Objectives

The primary and secondary objectives of the LIGHT-ACD trial are to compare blood pressure changes and blood pressure control between different initial antihypertensive regimens, respectively.

#### Trial design and setting

The LIGHT-ACD trial is an individually randomized trial conducted in the intervention sites of the LIGHT trial. In this nested trial, patients are randomized to receive various initial antihypertensive therapies, and blood pressure changes/control rates between different regimens are compared (Fig. 1).

#### Recruitment of patients

The patients at the intervention sites of the LIGHT trial are eligible if they have a measured baseline SBP ≥140 mmHg and are taking 0–1 class of antihypertensive medication. Key exclusion criteria include diabetes mellitus and intolerance to at least one of the four classes of antihypertensive medications (Table 1). The eligible patients in the LIGHT-ACD trial are categorized into 2 subpopulations. Patients with an SBP of 140–159 mmHg who are not taking any antihypertensive medication are categorized as Population 1, the remainder as Population 2.

#### Randomization and blinding

Populations 1 and 2 are randomized to different antihypertensive regimens separately. Minimization randomization [26] is used to ensure the balancing of age, gender, and education level among different arms. The allocation is centrally performed and concealed within the CDSS. Neither doctors nor patients are blinded to treatment allocation. Population 1 individuals are randomized to receive one of the initial monotherapies of A (ACEI or ARB), C (CCB), or D (diuretics). If additional treatment is needed, they are further randomized to add either C or D, so patients initiated with A follow either A-AC-ACD or A-AD-ADC with C and D as the add-on medications, respectively. The randomization of drug regimen among patients initiated with C or D is similar to patients initiated with A. Population 2 individuals are randomized to receive one of the three initial dual therapies of AC, AD, or CD. Subsequently, D, C, or A are added to achieve blood pressure control, if necessary, respectively (Fig. 1).

#### Treatments

The assignment of treatment is presented as the CDSS recommendation (class and dose). The specific agent within each class is at the doctor’s discretion based on the available medications of the sites. For each case, the titration of antihypertensive medication is performed automatically by the CDSS according to the assigned drug regimens.

#### Outcomes

The primary outcome is the difference in the change in blood pressure from baseline to 9 months between different drug regimens of initial therapy. Secondary outcomes include the proportion of individuals with blood pressure controlled at 9 months; the proportion of individuals with SBP <160 mmHg and DBP <100 mmHg at 9 months; the proportion of individuals who received monotherapy (only in Population 1), dual therapy, triple therapy, and referral at 9 months; the proportion of individuals reported to have antihypertensive drug-related side-effects; and the proportion of individuals transferred to usual care for any reasons. The exploratory outcome is the change in blood pressure from baseline to 9 months of six protocols embedded in the CDSS (Table 2).

#### Sample size

We assume approximately 25% of the LIGHT intervention patients are in Population 1 and 75% in Population 2, with an 80% follow-up rate for the primary outcome. We used estimates from the ALLHAT (Antihypertensive and Lipid-Lowering Treatment to Prevent Heart Attack Trial) for power calculations [18, 27]. For each population, we estimate the detectable difference in SBP between treatment groups across a similar range of the intervention participants and statistical power. We assume that the standard deviation in SBP is *σ* = 10 mmHg and that the within-patient SBP correlation is *R*^2^ = 0.2 with a maximum type I error that is Sidak-corrected for three comparisons, *α* = 0.017 [28]. With the current number of LIGHT sites, we estimate about 2100 eligible LIGHT-ACD participants overall with complete follow-up. Under these assumptions, we estimate that for the comparisons of initial monotherapies of A, C, and D in Population 1, we will have 80% power to detect a difference of 3.5 mmHg in SBP, and for comparisons of dual therapies of AC, AD, and CD in Population 2, we have 80% power to detect a difference of 2 mmHg in SBP.

#### Statistical analysis

All the intervention evaluations will be performed on an intention-to-treat basis. Multiple imputations by chained equations will be used to account for missing values.

Patient characteristics will be summarized by comparison group and tested for differences using *t*-tests and chi-square tests. To assess the effectiveness of each initial antihypertensive regimen and protocol, we will estimate a mixed-effects generalized linear model for each outcome including indicators for treatment groups, baseline blood pressure, and imbalanced patient characteristics. Additionally, we will perform pre-specified subgroup analyses of outcomes by age, sex, education, smoking status, and tertiles of baseline blood pressure. Considering potential crossovers among treatments, we will also assess the heterogeneity of the treatment effect among per-protocol populations.

## Discussion

The LIGHT trial is, to the best of our knowledge, the largest pragmatic randomized trial exploring the feasibility and effectiveness of a decision support tool to deliver high-quality care for hypertension in primary care. Moreover, by adopting a streamlined study design, we have nested a patient-level randomized trial (LIGHT-ACD trial) into this cluster-randomized trial using an algorithm-based CDSS tool.

Our studies have several potential strengths. First, we have developed a usable CDSS, which can easily be integrated into the routine clinical workflow and provides tailored antihypertensive recommendations at the point of care. These features are highly correlated with the effectiveness of CDSS for improving the process of care and patient outcomes [23, 29, 30]. As recommendations of CDSS are generated automatically by the built-in algorithm, which is developed based on current guidelines, this approach can assist primary care doctors, even those with less training, in making informed and evidence-based prescribing decisions.

Second, we have built a streamlined framework for a clinical trial that enabled us to compare the effectiveness of several guideline-based initial antihypertensive regimens. Earlier randomized clinical trials such as the ALLHAT [18] and ACCOMPLISH [31] trials provided a direct comparison among several monotherapies or dual therapies. These trials are not contemporary and did not include large Asian populations, so there remain gaps in knowledge. In contrast with these standalone trials, the conduct of the LIGHT-ACD trial is embedded into the existing framework of the LIGHT trial. We incorporate a series of stepped treatment protocols into the CDSS, whereby the random allocation of recommended medication can be performed automatically by following the algorithm-consistent order at each encounter where decision support is delivered [32]. While assessing the effectiveness of CDSS, the effectiveness of common initial antihypertensive monotherapies or dual therapies can be compared unobtrusively [31, 33–35].

Third, the pragmatic design of both trials enables us to demonstrate the real-world effectiveness of intervention/treatment with greater external validity [36, 37]. In contrast to trials with study-specific visits, the enrolment and follow-up of patients, and the collection of outcome data in our trial are incorporated into routine clinical practice. Furthermore, the exclusion criteria are kept to a minimum to enroll a diverse spectrum of the population. These considerations improve the efficiency of trials and enhance the generalizability of the study results [37].

Fourth, the two studies are further distinguished by the efforts to build a learning decision support tool. Although the algorithm of CDSS is kept consistent during the four stages of the trial, it has the potential to be adaptively updated after the results of the LIGHT-ACD trial are available. For the ultimate implementation of the CDSS, the tool could continuously generate new knowledge in terms of the effectiveness of treatment strategies from the ongoing delivery of care, whereby the CDSS could be iteratively tested and improved by shifting the randomization ratio of stepped antihypertensive protocols toward the more effective group [38].

Our study has some potential limitations. First, the outcomes are focused on surrogate outcomes and not clinical outcomes such as cardiovascular events. To examine the effect on clinical events, a much longer trial would be required. However, it is expected that improvements in blood pressure control over time would favorably affect clinical outcomes. Second, given the nature of the CDSS, which delivers its recommendation directly to doctors, blinding was not feasible. We minimized the potential bias by using objective measures as primary and secondary outcomes. Third, due to the limited timeframe of the study, an extended follow-up was not included following the intervention to measure the persistence of effects after the intervention ceases. Fourth, we did not include consideration of diet, in part because of issues of the validity of self-reporting. In this trial, we only collected information necessary for pharmacological hypertension management such as the blood pressure measurements, comorbidities, current medications, and prescriptions. However, the confounding effect of diet may have been minimized through site randomization.

In conclusion, both trials should be able to provide useful evidence regarding the effectiveness of this decision support tool on improving adherence to guidelines for hypertension management in primary care, and on the comparative effectiveness of different initial antihypertensive regimens for blood pressure reduction in the real-world setting.

### Trial status

Protocol version 4.4 commenced on December 28, 2020. As of May 2021, 94 sites (including 7,656 patients in LIGHT and 494 participants in the LIGHT-ACD trial) have been randomized in 4 stages. Site randomization of Stage 1 was on 21 August 2019, Stage 2 on 4 December 2019, Stage 3 on 1 March 2021, and Stage 4 on 14 April 2021. The implementation of the trial is affected by the outbreak of COVID-19, we have extended the enrolment and follow-up periods appropriately in each stage of the trial. Currently, all sites will complete follow-up by the middle of 2022.

## Supplementary Information

**Additional file 1: Supplement 1.** Stratification factors for each stage in site randomization of LIGHT trial.

**Additional file 2: Supplement 2.** Specification of hypertension visits.

**Additional file 3: Supplement 3.** Example of CDSS recommendation.

**Additional file 4: Supplement 4.** Specifications of guideline-based antihypertensive treatment.

**Additional file 5: Supplement 5.** Trial registration data of the LIGHT trial.

**Additional file 6: Supplement 6.** Trial registration data of the LIGHT-ACD trial.

## Data Availability

The datasets used and/or analyzed during the current study will be available from the corresponding author on reasonable request.
