# Supplementary material for: Effectiveness of a clinical decision support system for hypertension management in primary care: study protocol for a pragmatic cluster-randomized controlled trial
Source: Trials. 2022 May 16;23:412. doi: 10.1186/s13063-022-06374-x (PMC9109449; doi:10.1186/s13063-022-06374-x)
Supplement: Supplementary file 1 — Additional file 1: Supplement 1. Stratification factors for each stage in site randomization of LIGHT trial. [file 13063_2022_6374_MOESM1_ESM.docx]

**Supplement 1.** Stratification factors for each stage in site randomization of LIGHT trial

1. Stage 1 (18 sites in Luoyang, Henan Province): baseline appropriate antihypertensive treatment rates of the site (≥median/<median) and the hospital to which the site is affiliated (Dongfang Hospital/Sixth People’s Hospital/Zhongxin Central Hospital).
2. Stage 2 (9 sites in Zoucheng, Shandong Province): baseline appropriate antihypertensive treatment rates of the site (≥median/<median) and type of primary care practice (Fenyuan/Community health service station).
3. Stage 3 (31 sites in Shenzhen, Guangdong Province): baseline appropriate antihypertensive treatment rates of the site (≥median/<median) and geographical region (Baoan District/Nanshan District).
4. Stage 4 (36 sites in Shenzhen, Guangdong Province): baseline appropriate antihypertensive treatment rates of the site (≥median/<median) and geographical region (Luohu District/Longgang District/Futian District).
