# Supplementary material for: Effectiveness of a clinical decision support system for hypertension management in primary care: study protocol for a pragmatic cluster-randomized controlled trial
Source: Trials. 2022 May 16;23:412. doi: 10.1186/s13063-022-06374-x (PMC9109449; doi:10.1186/s13063-022-06374-x)
Supplement: Supplementary file 2 — Additional file 2: Supplement 2. Specification of hypertension visits. [file 13063_2022_6374_MOESM2_ESM.docx]

**Supplement 2.** Specification of hypertension visits

1. Scheduled or unscheduled visits for treatment of hypertension
2. Visit for diabetes, stroke, peripheral vascular disease, or other new cardiovascular diseases (i.e., chronic kidney disease, coronary heart disease, and heart failure).
