# Supplementary material for: Effectiveness of a clinical decision support system for hypertension management in primary care: study protocol for a pragmatic cluster-randomized controlled trial
Source: Trials. 2022 May 16;23:412. doi: 10.1186/s13063-022-06374-x (PMC9109449; doi:10.1186/s13063-022-06374-x)
Supplement: Supplementary file 3 — Additional file 3: Supplement 3. Example of CDSS recommendation. [file 13063_2022_6374_MOESM3_ESM.docx]

**Supplement 3.** Example of CDSS recommendation

“The recommended antihypertensive medications are C+D

C (calcium channel blocker): full dose

D (diuretics): half dose”

Note: Full dose or half dose refer to one or half pill of commonly used antihypertensive medications respectively.
