# Supplementary material for: Effectiveness of a clinical decision support system for hypertension management in primary care: study protocol for a pragmatic cluster-randomized controlled trial
Source: Trials. 2022 May 16;23:412. doi: 10.1186/s13063-022-06374-x (PMC9109449; doi:10.1186/s13063-022-06374-x)
Supplement: Supplementary file 4 — Additional file 4: Supplement 4. Specifications of guideline-based antihypertensive treatment. [file 13063_2022_6374_MOESM4_ESM.docx]

**Supplement 4.** Specifications of guideline-based antihypertensive treatment

1. Uptitrating or switching treatment for patients with inadequate blood pressure control.
   - Uptitrating or switching treatment for patients who: a) have systolic blood pressure (SBP) 140**–**159 mmHg or diastolic blood pressure (DBP) 90–99 mmHg; AND b) have established diagnosis of hypertension less than 3 months; AND c) have complications of hypertension (i.e., diabetes, chronic kidney diseases, stroke, myocardial infarction, and heart failure)
   - Uptitrating or switching treatment for patients who: a) have SBP 140**–**159 mmHg or DBP 90–99 mmHg; AND b) have established diagnosis of hypertension more than 3 months; AND c) take no antihypertensive medications.
   - Uptitrating or switching treatment for patients who: a) have SBP ≥160 mmHg or DBP ≥100 mmHg; AND b) take no antihypertensive medications.
   - Uptitrating or switching treatment for patients who: a) have SBP ≥140 mmHg or DBP ≥90 mmHg; AND b) take at least one antihypertensive medication.
2. Antihypertensive medications used in patients with specific clinical indications for their use.
   - Use of beta-blockers for patients with myocardial infarction and heart failure.
   - Use of angiotensin-converting enzyme inhibitors (ACEI) or angiotensin receptor blockers (ARB) for patients with diabetes, chronic kidney disease, myocardial infarction, and heart failure.
3. Antihypertensive medications used in patients without compelling contraindications for their use.
   - Use of diuretics for patients without gout.
   - Use of beta-blockers for patients without bradycardia (heart rate <50 beats/min).
   - Use of angiotensin-converting enzyme inhibitors (ACEI) for patients without hyperkalemia (potassium >5.5 mmol/L) or previous angioneurotic edema.
   - Use of angiotensin receptor blockers (ARB) for patients without hyperkalemia (potassium >5.5 mmol/L).
4. Antihypertensive medications used in patients without intolerance for their use
   - Use of calcium channel blockers (CCB) for patients without intolerance (e.g., CCB-induced edema) to their use
   - Use of ACEI for patients without intolerance (e.g., ACEI-induced cough) to their use
5. Use of guideline-based antihypertensive medication
   - Any antihypertensive medications beyond ACEI, ARB, beta-blocker, CCB, and diuretics not used for patients with complications.
   - Any antihypertensive medications beyond ACEI, ARB, beta-blocker, CCB, diuretics, compound reserpine triamterene, and compound reserpine) not used for patients without complications.
6. Other
   - Agents within the same class of antihypertensive medications not used at the same time.
   - Referral for patients with full dose of ACEI/ARB, CCB, and diuretics but with inadequate blood pressure control.
   - Single use of short-acting antihypertensive medications with frequency compliance to that of specified in the instruction
