# Supplementary material for: Effectiveness of a clinical decision support system for hypertension management in primary care: study protocol for a pragmatic cluster-randomized controlled trial
Source: Trials. 2022 May 16;23:412. doi: 10.1186/s13063-022-06374-x (PMC9109449; doi:10.1186/s13063-022-06374-x)
Supplement: Supplementary file 5 — Additional file 5: Supplement 5. Trial registration data of the LIGHT trial. [file 13063_2022_6374_MOESM5_ESM.docx]

**Supplement 5.** Trial registration data of the LIGHT trial

| 1. Primary Registry and Trial Identifying Number | ClinicalTrials.gov NCT03636334 (LIGHT) |
| --- | --- |
| 2. Date of Registration in Primary Registry | 3 July 2018 |
| 3. Secondary Identifying Numbers | Not applicable |
| 4. Source(s) of Monetary or Material Support | CAMS Innovation Fund for Medical Science (2016-I2M-1-006) |
| 5. Primary Sponsor | Chinese Academy of Medical Sciences and Peking Union Medical College |
| 6. Secondary Sponsor(s) | Not applicable |
| 7. Contact for Public Queries | **Professor Xin Zheng**  National Clinical Research Center for Cardiovascular Diseases, State Key Laboratory of Cardiovascular Disease, Chinese Academy of Medical Sciences and Peking Union Medical College, Fuwai Hospital, National Center for Cardiovascular Diseases, Beijing, China  xin.zheng@fwoxford.org |
| 8. Contact for Scientific Queries | **Professor Xin Zheng**  National Clinical Research Center for Cardiovascular Diseases, State Key Laboratory of Cardiovascular Disease, Chinese Academy of Medical Sciences and Peking Union Medical College, Fuwai Hospital, National Center for Cardiovascular Diseases, Beijing, China  xin.zheng@fwoxford.org |
| 9. Public Title | Rationale and design of the Learning Implementation of Guideline-based decision support system for Hypertension Treatment (LIGHT) Trial and LIGHT-ACD Trial |
| 10. Scientific Title | Rationale and design of the Learning Implementation of Guideline-based decision support system for Hypertension Treatment (LIGHT) Trial and LIGHT-ACD Trial |
| 11. Countries of Recruitment | China |
| 12. Health Condition(s) or Problem(s) Studied | Hypertension |
| 13. Intervention(s) | Intervention arm: computerized clinical decision support systems for hypertension management  Control arm: usual care |
| 14. Key Inclusion and Exclusion Criteria | See main body of protocol |
| 15. Study Type | A pragmatic, parallel-group, four-stage, cluster-randomized controlled trial using stratified randomization |
| 16. Date of First Enrolment | 21 August 2019 |
| 17. Target Sample Size | 94 sites |
| 18. Recruitment Status | Recruiting |
| 19. Primary Outcome(s) | Proportion of hypertension visits during which appropriate antihypertensive treatment is prescribed |
| 20. Key Secondary Outcomes | Average change in systolic blood pressure, the proportion of those whose blood pressure is controlled at 9 months, and the proportion of hypertension visits with acceptable antihypertensive treatment |
