# Supplementary material for: Effectiveness of a clinical decision support system for hypertension management in primary care: study protocol for a pragmatic cluster-randomized controlled trial
Source: Trials. 2022 May 16;23:412. doi: 10.1186/s13063-022-06374-x (PMC9109449; doi:10.1186/s13063-022-06374-x)
Supplement: Supplementary file 6 — Additional file 6: Supplement 6. Trial registration data of the LIGHT-ACD trial. [file 13063_2022_6374_MOESM6_ESM.docx]

**Supplement 6.** Trial registration data of the LIGHT-ACD trial

| 1. Primary Registry and Trial Identifying Number | ClinicalTrials.gov NCT03587103 (LIGHT-ACD) |
| --- | --- |
| 2. Date of Registration in Primary Registry | 3 July 2018 |
| 3. Secondary Identifying Numbers | Not applicable |
| 4. Source(s) of Monetary or Material Support | CAMS Innovation Fund for Medical Science (2016-I2M-1-006) |
| 5. Primary Sponsor | Chinese Academy of Medical Sciences and Peking Union Medical College |
| 6. Secondary Sponsor(s) | Not applicable |
| 7. Contact for Public Queries | **Professor Xin Zheng**  National Clinical Research Center for Cardiovascular Diseases, State Key Laboratory of Cardiovascular Disease, Chinese Academy of Medical Sciences and Peking Union Medical College, Fuwai Hospital, National Center for Cardiovascular Diseases, Beijing, China  xin.zheng@fwoxford.org |
| 8. Contact for Scientific Queries | **Professor Xin Zheng**  National Clinical Research Center for Cardiovascular Diseases, State Key Laboratory of Cardiovascular Disease, Chinese Academy of Medical Sciences and Peking Union Medical College, Fuwai Hospital, National Center for Cardiovascular Diseases, Beijing, China  xin.zheng@fwoxford.org |
| 9. Public Title | Rationale and design of the Learning Implementation of Guideline-based decision support system for Hypertension Treatment (LIGHT) Trial and LIGHT-ACD Trial |
| 10. Scientific Title | Rationale and design of the Learning Implementation of Guideline-based decision support system for Hypertension Treatment (LIGHT) Trial and LIGHT-ACD Trial |
| 11. Countries of Recruitment | China |
| 12. Health Condition(s) or Problem(s) Studied | Hypertension |
| 13. Intervention(s) | Population 1: Initial monotherapy of A, C, and D  Population 2: Initial dual-therapy of AC, AD, and CD |
| 14. Key Inclusion and Exclusion Criteria | See main body of protocol |
| 15. Study Type | A pragmatic, three-arm, patient-randomized trial using minimization randomization |
| 16. Date of First Enrollment | 21 August 2019 |
| 17. Target Sample Size | 2100 patients |
| 18. Recruitment Status | Recruiting |
| 19. Primary Outcome(s) | Change in blood pressure from baseline to 9 months |
| 20. Key Secondary Outcomes | Proportion of individuals with blood pressure controlled at 9 months; the proportion of individuals with SBP <160 mmHg and DBP <100 mmHg at 9 months |
